# Sirolimus Treatment for Complex Fetal and Neonatal Vascular Anomalies: Emerging Evidence and Safety Considerations

**DOI:** 10.3390/jcm15103739

**Published:** 2026-05-13

**Authors:** Elena Țarcă, Elena Cojocaru, Lăcrămioara Ionela Butnariu, Solange Tamara Roșu, Alina Costina Luca, Sidonia Susanu, Vasile Valeriu Lupu, Eduard Vasile Roșu, Paula Popovici, Viorel Țarcă

**Affiliations:** 1 Grigore T. Popa University of Medicine and Pharmacy, 700115 Iasi, Romania; tarca.elena@umfiasi.ro (E.Ț.); elena2.cojocaru@umfiasi.ro (E.C.); ionela.butnariu@umfiasi.ro (L.I.B.); alina.luca@umfiasi.ro (A.C.L.); vasile.lupu@umfiasi.ro (V.V.L.); eduard.rosu@umfiasi.ro (E.V.R.); paula.popovici@umfiasi.ro (P.P.); 2 Department of Plastic Surgery, Saint Mary Emergency Children Hospital, 700309 Iași, Romania; s.susanu@gmail.com; 3 Faculty of Medicine, Apollonia University, Strada Păcurari nr. 11, 700511 Iași, Romania; viorel.tarca@univapollonia.ro

**Keywords:** vascular abnormalities, neonate, rapamycin, sirolimus treatment

## Abstract

**Background**: Vascular abnormalities (VAs) are a broad category of disorders that include vascular malformations (inadequate embryologic blood vessel development) and vascular tumors (characterized by vascular cell hyperplasia). This sometimes-complicated pathology is increasingly being treated with mTOR inhibitors, particularly sirolimus (rapamycin). Only a limited number of neonates treated with sirolimus have been reported in the literature to date, and there is no consensus regarding the optimal treatment regimen for vascular anomalies in this population. The objective of our narrative review is to summarize the most recent information from the specialized literature regarding the efficacy and safety of sirolimus in newborns. **Methods**: A methodological search was performed through the available data concerning the indications and safety when using sirolimus to treat VAs in newborns. We evaluated articles from the Embase and PubMed academic search engines using the following search terms: (vascular anomalies OR vascular tumors OR venous malformations OR lymphatic malformations OR capillary malformations) AND (neonate) AND (sirolimus OR rapamycin). **Results**: We identified a lack of consensus regarding indications for treatment initiation, optimal dosing regimens, and duration of therapy. In most published studies, neonates were analyzed within broader pediatric cohorts that included patients up to 18 years of age, rather than as a distinct population. Moreover, only a limited number of investigations have specifically focused on neonates treated with sirolimus and evaluated outcomes from a neonatal subgroup perspective. The available evidence largely consists of isolated case series and reports describing sirolimus use in fetuses and neonates, most commonly for lymphatic malformations or kaposiform hemangioendothelioma; in some instances, sirolimus was administered in combination with propranolol, corticosteroids, or other therapeutic modalities. Nevertheless, across studies and case reports involving fetuses or neonates, sirolimus is generally reported to be effective and well tolerated, with adverse effects that are minimal and reversible. **Conclusions**: For neonates and even fetuses with large, complex VAs, sirolimus appears to be an effective treatment with no serious adverse events reported to date. Decisions regarding off-label sirolimus initiation should be made by a multidisciplinary team, and parents must be thoroughly informed about potential adverse events. Well-designed randomized controlled trials or high-quality observational studies are needed to further evaluate the efficacy and safety of sirolimus in the neonatal population.

## 1. Introduction

Vascular malformations (inadequate embryologic blood vessel development) and vascular tumors (defined by vascular cell hyperplasia) comprise the diverse category of conditions known as vascular abnormalities (VAs) [1]. An updated classification system was approved by the International Society for the Study of Vascular Anomalies (ISSVA) in May 2024 at the ISSVA World Congress in Madrid, Spain, and was further revised based on feedback until 2025 [2].

Vascular malformations have been linked to several somatic, pathogenic activating variations in the genes involved in angiogenesis and lymphangiogenesis, including TEK (OMIM 600221) and PIK3CA (OMIM 171834) [3]. A variety of developmental phenotypes, including vascular malformations, megalencephaly–capillary malformation (MCAP) syndrome, congenital lipomatous overgrowth, epidermal nevi, scoliosis/skeletal and spinal (CLOVES) syndrome, and fibroadipose overgrowth, have been linked to postzygotic activating mutations of PIK3CA [4]. PIK3CA mutations may also be the cause of some Klippel–Trenaunay syndrome (KTS) cases [5,6]; on the other hand, TEK variations are more frequently discovered in venous malformations [7]. Some VAs can endanger the patient’s life, even from the moment of birth. Clinical issues include respiratory insufficiency, deformity, persistent discomfort, recurrent infections, thrombotic and hemorrhagic coagulopathies, organ malfunction, and death that can result from the growth and/or extension of VAs.

This complicated pathology is increasingly being treated with mTOR inhibitors, particularly sirolimus (rapamycin), which was typically prescribed as an immunosuppressive medication to prevent organ rejection [8]. Sirolimus directly inhibits mTOR, which is regulated by phosphoinositide-3-kinase and functions as a master switch in various physiological processes, including angiogenesis, lymphangiogenesis, cell growth, and proliferation [5]. Sirolimus also helps control the inflammatory response linked to venous or lymphatic malformations by further reducing inflammatory cell activation and cytokine release [9]. It is gradually becoming the accepted standard molecular treatment for resistant malformation lesions in adults and children, according to Seront et al. [10].

The Observational-Phase Randomized Clinical PERFORMUS Trial, a prospective study conducted in 2021, indicated significant reductions in complicated vascular anomalies and superficial slow-flow vascular malformations among children and young people treated with rapamycin [3]. In January 2024, Cavazos et al. published a systematic review about the efficacy and safety of sirolimus in treating congenital vascular anomalies in the first year of life. There were 172 participants in 84 case series and reports. In more than half of patients, sirolimus reduced the size of the VA; the majority experienced mild, temporary side effects, and 27% experienced no adverse effects [11].

However, only a limited number of neonates treated with sirolimus have been reported in the literature to date, and there is no consensus regarding the optimal treatment regimen for vascular anomalies in this population. Moreover, therapeutic alternatives during the neonatal period are extremely limited, and the efficacy of these options has not been confirmed in prospective clinical trials [12]. The type of malformation, the localization, its symptoms, and its complications all influence the treatment options. Unfortunately, some VAs developed during fetal life can endanger the life of the newborn, their treatment constituting a neonatal emergency. Therefore, this gap between the urgent treatment of vascular anomalies in neonates and the safe therapeutic implementation of an off-label medication like sirolimus represents a critical unmet need in neonatal VAs management.

The objective of our narrative review is to summarize the most recent information from the specialized literature concerning the efficacy and safety of sirolimus to treat vascular abnormalities in newborns.

## 2. Materials and Methods

The paper was prepared using the SANRA checklist (Scale for the Assessment of Narrative Review Articles), and despite not being systematic, a methodological search was carried out throughout the review process to ensure the quality of the manuscript [13]. Four members of our research team carried out independent data extraction and quality assessment as part of the strategy, each of whom contributed to the review’s accuracy and comprehensiveness.

### 2.1. Search Strategy of Electronic Databases

Between 1 November 2025, and 30 December 2025, we reviewed the available data regarding the use of sirolimus to treat VAs in newborns. We evaluated articles from the Embase and PubMed databases using the following search terms: (vascular anomalies OR vascular tumors OR venous malformations OR lymphatic malformations OR capillary malformations) AND (neonate) AND (sirolimus OR rapamycin). The primary goal of the search was to find original studies, reviews, or case series that discussed the use of sirolimus for vascular anomalies in young children, particularly in fetuses and neonates, with an emphasis on the drug’s effectiveness, safety, or adverse effects.

#### 2.1.1. Including and Excluding Criteria

The following criteria were met by the studies included in this review to ensure the inclusion of the most pertinent and high-quality evidence: full-text availability online, publication within the previous ten years, a clearly stated methodology, human subjects, and English-language articles. Randomized controlled trials, observational and clinical studies, reviews, and case studies were all included in our search. Depending on the particularities of each of the two databases searched, we applied additional filters to find the most relevant articles on the specified topic.

To provide a thorough investigation of the indication of sirolimus administration, the drug’s efficacy, safety, and adverse effects on young children and neonates with VAs, a wide range of data, from extensive quantitative analyses to in-depth reports of individual patient experiences, were analyzed. To find other potentially pertinent papers, we looked through the reference lists of the included studies. Studies that were redundant, irrelevant, or used non-human subjects were excluded.

#### 2.1.2. Study Selection and Information Extraction

The publications included in the review were selected by four authors using database searches. They independently verified the eligibility of the abstract and title of each selected study. If there was disagreement among the reviewers, a fifth author acted as a referee; otherwise, a consensus was established. Duplicate references and non-human research were excluded. Two additional authors examined the references of the pertinent publications to identify appropriate research that would meet inclusion requirements. By following this strategy, we intended to present an unbiased, thorough, and current synthesis of the literature on the use of sirolimus to treat vascular abnormalities in neonates, offering useful information about this difficult area of pediatric medicine.

## 3. Results

The initial PubMed search retrieved a total of 105 results for all years and 94 from the last 10 years. After applying the filters (full-text available online, English language, humans, age: newborn: birth-1 month), 19 studies remained to be screened for eligibility.

The initial Embase search retrieved a total of 182 results for all years and 171 from the last 10 years. After applying the filters: publication types (articles, clinical trials, reviews), human, age (‘fetus’ OR ‘newborn’) and diseases (‘lymphatic malformation’ OR ‘blue rubber bleb nevus’ OR ‘congenital blood vessel malformation’ OR ‘cystic lymphangioma’ OR hemangioendothelioma’ OR ‘hemangioma’ OR ‘kaposiform hemangioendothelioma’ OR ‘kasabach merritt syndrome’ OR ‘vascular tumor’), 101 studies remained to be screened for eligibility.

During the second step, the authors reviewed the titles, abstracts, and full texts of the papers and selected the most relevant studies reporting sirolimus/rapamycin treatment in neonates, the efficacy, safety, and long-term results. A total of 50 publications is included in our reference list.

## 4. Vascular Anomalies and Sirolimus Treatment

A multidisciplinary approach and customized treatment based on symptoms, functional impairment, lesion size, type, and location are frequently required for the management of VAs. Traditionally, sclerotherapy, corticosteroids, vincristine, compression therapy, laser therapy, and surgery have all been used to address this pathology, each presenting specific limitations [9]. While laser therapy works well for tiny, superficial venous or capillary malformations, it is less successful for large or deep VAs and might cause pigmentation or skin burns. Surgery is a good option for treatment for children and adults with complex VAs, such as those with microcystic and mixed lymphatic malformations (LMs). In cases of macrocystic LMs, sclerotherapy works very well as an adjuvant treatment when combined with surgery. Although there were several potential sclerosants, absolute ethanol, bleomycin, doxycycline, and picibanil (OK-432) are the most commonly utilized [14]. They are tissue irritants that damage and inflame the endothelium, resulting in vascular obliteration and fibrosis. The subsequent inflammatory response may cause discomfort and be hazardous (airways, eyes). Efficacy is assessed after a minimum of four to six weeks, and more sessions may be required. Compression therapy mostly helps reduce symptoms in extremities affected by lymphatic or venous abnormalities, but fails to reduce the malformation size or stop its progression. However, when it comes to the urgent treatment of a life-threatening vascular anomaly in a newborn, sclerotherapy is only an adjuvant solution at most, as immediate therapy to reduce and stop angiogenesis, lymphangiogenesis, cell growth, and proliferation is necessary.

Rapamycin (sirolimus) was initially identified in a Rapa Nui (Easter Island) soil sample. The soil bacterium *Streptomyces hygrosopicus* is the source of this naturally occurring macrolide. In 1999, the U.S. Food and Drug Administration (FDA) approved sirolimus as an oral immunosuppressive for use in renal transplantation. In 2015, the FDA approved sirolimus as the first medication to treat lymphangioleiomyomatosis, a rare disease that primarily affects young women and is characterized by progressive cystic lung disease [15]. Since 2015, sirolimus has been increasingly used in the treatment of vascular abnormalities such as hemangiomas, Kaposi hemangioendotheliomas (KHE), and lymphatic and venous malformations in children, neonates and even in fetuses. Its application in the treatment of arteriovenous malformations in neonates and small children has rarely been documented [16]. A prospective phase II trial currently investigates the effectiveness of sirolimus in arteriovenous malformations [17].

### 4.1. Slow-Flow Malformations

Capillary malformations, venous malformations (VMs), lymphatic malformations (LMs), or combinations of these anomalies are examples of slow-flow vascular malformations, according to ISSVA Classification 2025 [2].

#### 4.1.1. Lymphatic Malformations

Although the exact cause of LMs is yet unknown, it is generally accepted that they are aberrant, non-malignant lymphatic vessel formation caused by congenital, sporadic, developmental disorders that occur in the early stages of embryogenesis. LMs are classified as macrocystic (>1 cm), microcystic (<1 cm), or mixed lesions based on cyst size, with an estimated prevalence of 1 in 2000 newborns [2,15]. While large LMs can also be identified prenatally (ultrasonography and/or MRI) and some LMs have delayed manifestation in adulthood, LMs are typically diagnosed at birth or within the first two years of life. Although a biopsy is rarely required to diagnose LMs, it can be utilized in conjunction with a blood sample for molecular biology tests to look for activating mutations of the PIK3CA gene, especially when LMs are integrated in a syndromic form, including Proteus, Klippel–Trenaunay, and CLOVES syndrome. Symptoms in the head and neck region can vary from mild swelling to potentially fatal airway blockage at birth, poor oral feeding, macroglossia, mandibular overgrowth, vision loss, discomfort, and cosmetic deformity. In the event of a predictable obstruction during childbirth, an Ex Utero Intrapartum Treatment (EXIT) intervention is recommended. Prenatal consultations should address the likelihood that a tracheotomy may be required [18].

Treatment options for life-threatening, obstructive lymphatic malformations in newborns are limited and include surgery, sclerotherapy, and corticotherapy, all of which carry considerable risks for the newborn. Because sirolimus is a feasible treatment with acceptable safety, Honnorat et al. propose that it be used as an adjuvant therapy in the first line for newborns with lymphatic abnormalities. This is especially interesting for preterm infants, who may experience serious problems from surgery and sclerotherapy operations. When the location (larynx, base of the tongue) and/or the microcystic type are difficult to eradicate with surgery or sclerotherapy without adverse effects, this therapy may also be considered [19]. Combining sirolimus (for 12–24 months) with one or more sclerotherapy procedures has shown very positive outcomes in large LMs [20]. A systematic review published in 2022 analyzed 28 studies encompassing 105 pediatric patients with head and neck LMs and identified only 16 neonates who had been treated with sirolimus up to that time [15]. The most commonly reported initial dosage was 0.8 mg/m^2^ administered twice daily at 12 h intervals. Although target therapeutic blood concentrations varied across studies, the most frequently cited ranges were 10–15 ng/mL and 5–15 ng/mL. More than 91% of patients demonstrated a clinical response to sirolimus therapy, with treatment durations ranging from six months to four years. Notably, a neonate who initiated sirolimus therapy on the 15th day of life and continued treatment for one year achieved complete remission of a cervicofacial macrocystic LM, with no recurrence observed after one year of follow-up [21].

In a recent study of 56 patients with intra-abdominal LMs, thirteen cases (23%) were present at birth. At some time throughout their clinical course, sirolimus was administered to twenty-two patients (39% of the total), playing an important role in the multi-modal treatment of patients with PROS. Although the goal of the sirolimus-treated patients’ therapy was to alleviate their overall symptoms, it was challenging to determine with certainty whether these patients needed fewer surgical or medical interventions [22].

Sirolimus was even administered as prenatal treatment for massive LM discovered in utero, with encouraging results that require confirmation [23,24]. In a study published in 2024, five neonates treated with EXIT for prenatally diagnosed cervicofacial LMs beneficiated of sirolimus treatment, according to the recent paradigm shift in postnatal management of cervicofacial LMs from resection alone toward a multimodal approach including sirolimus and sclerotherapy. This change of paradigm reflects a growing knowledge of the pathophysiology and natural history of lymphatic anomalies as well as an awareness of the limitations of surgical procedures, such as recurrence and inadequate excision. According to the authors, starting sirolimus is becoming a common part of the postnatal treatment plan for cervicofacial LMs [25]. According to Strychowsky et al. [26], younger patients may respond better to sirolimus than older patients, and macrocystic LM may respond better than mixed or microcystic LM.

#### 4.1.2. Venous Malformations (VMs)

Venous malformations are the most prevalent kind of congenital vascular abnormalities that affect roughly 1% of the general population and occur in 1 to 2 out of every 10,000 newborns [9]. Inflammatory processes, genetic mutations, and structural abnormalities are their defining characteristics. The venous walls in these abnormalities are considerably weaker than normal, mostly due to reduced elastic fibers and smooth muscle cells, coupled with deficiencies in connective tissue components. There are many current treatment strategies for VMs, each presenting specific limitations. When treating large or complicated venous malformations that cannot be treated with surgery or other physical therapies, sirolimus is especially useful [12].

Wang et al. examined eight clinical studies involving 74 patients with VMs to evaluate the efficacy and safety of sirolimus for treating these diseases. The data indicate that sirolimus dramatically reduces the size of VMs, raises hemoglobin levels, and improves coagulation function, consequently lowering the need for transfusions and substantially enhancing patients’ quality of life. Although mild to moderate side effects such as mouth ulcers and liver function abnormalities occurred, these were largely reversible following discontinuation or dose decrease [9]. Over the course of the 12-month research period, all parameters examined in pediatric patients with VMs showed notable improvement, and sirolimus was not linked to any serious adverse effects.

However, there are very few studies or reports of newborns with VMs treated with sirolimus. A recent case report describes a premature neonate from a twin pregnancy who required intubation and mechanical ventilation immediately after birth due to an extensive lymphatic–venous malformation involving the cervical and thoracic regions. Magnetic resonance imaging confirmed a complex and widespread lymphatic–venous malformation with a prominent venous component, compressing the airways. Following multidisciplinary discussion, the patient was treated with sirolimus and subsequently underwent limited surgical resection, lesion drainage, and vacuum-assisted closure of the left cervical lesion, resulting in resolution of the malformation [27].

The uncommon condition known as blue rubber bleb naevus syndrome (BRBNS) typically manifests as many venous abnormalities in the skin and digestive system. Due to its effect on angiogenesis signaling pathways, sirolimus has been successfully used to treat BRBNS in adults. Nevertheless, practitioners had little experience treating newborns with BRBNS. Though Yang et al. reported in 2021 a 38-day-old preterm female baby with skin lesions, hematochezia, and severe anemia. Hemoglobin was found to be 5.3 g/dL in the laboratory, and contrast-enhanced abdominal computed tomography revealed many low-density space-occupying lesions in the liver’s right lobe. Based on standard clinical and examination results, she was diagnosed with BRBNS. Despite receiving hemostatic medications and two transfusions, the patient’s anemia symptoms remained constant. The patient was then given sirolimus at a target medication level of 10–15 ng/mL at an average dose of 0.95 mg/m^2^/d. Hemoglobin levels recovered to normal, the lesion shrank, and there were no adverse drug reactions during the 28-month treatment course [28]. Another neonate with BRBNS and Kasabach–Merritt phenomenon (KMP) was successfully treated with oral low-dose sirolimus combined with glucocorticoids; the child’s lesions were considerably shrunk after six months of consistent monitoring, and there were no indications of a KMP recurrence [29].

#### 4.1.3. Combined Malformations

Congenital capillary–lymphatic–venous malformations (CLVMs) can cause life-threatening issues that start in utero. Very few cases of prenatal sirolimus treatment for fetal CLVMs with good outcomes have been reported to date, and there is a paucity of information on the optimal treatment regimen and dosage [23,30,31]. Klosowska et al. present two cases of successful prenatal and postnatal sirolimus treatment for large, complex fetal CLVMs. One of the pregnancies had polyhydramnios, and the other had intralesional hemorrhage. From the 32nd and 33rd weeks of pregnancy until birth, the expectant mothers took oral sirolimus (for 11 and 31 days, respectively) at doses ranging from 2 to 6 mg/day, with a target trough whole-blood level of 7–12 ng/mL. This led to umbilical cord arterial blood levels of 3.8 and 6.4 ng/mL, respectively. Both fetuses showed good therapeutic results from prenatal sirolimus: one had less intralesional hemorrhage, while the other had a marked reduction in CLVM size. Both infants, who are now 20 and 9 months old, have been receiving postnatal sirolimus medication. The treatment had no negative effects on the mothers or the children [30]. Repeated cordocentesis revealed a 30% transplacental crossing of sirolimus, according to Seront et al., who reported a successful management of a cervicofacial fetal LM with sirolimus taken orally by the mother from the 22nd week of pregnancy until two weeks before intended birth. After three weeks of sirolimus treatment and just one week after reaching the maternal target sirolimus blood level of 10–15 ng/mL, the lesion’s quick size decrease demonstrated the medication’s effectiveness [31]. Klippel–Trenaunay syndrome (KTS) is another rare congenital vascular disorder characterized by capillary malformations of the skin (port-wine stains), venous varicosities and venous/lymphatic malformations, and unilateral hypertrophy of bone and soft tissue, most commonly affecting the patient’s limb. No cure is available for KTS. Supportive care, treatment of comorbidities, orthopedic procedures for limb length discrepancy, and surgery to minimize tissue overgrowth are the key components of KTS management. Recent research, however, has shown that sirolimus works by inhibiting the mTOR protein, which contributes to the pathophysiology of KTS, making it a viable option for treating KTS, even in neonates [32]. Kuo et al. presented a neonatal case of KTS associated with chylothorax, with no response to standard treatment. They began giving sirolimus (0.8 mg/m^2^ twice daily) for the refractory chylothorax at 20 days of age. The patient responded when the sirolimus serum level reached the therapeutic range (10–15 ng/mL) at 26 days of age. After being discharged, he continued taking sirolimus, and as of the most recent follow-up in the outpatient department, at the age of seven months, chylothorax had not returned. Elevated gamma-glutamyl transferase was the only side effect of sirolimus medication, and there were no other frequently reported side effects [32].

### 4.2. Vascular Tumors and Sirolimus Treatment

Targeting the GLUT1 receptor, propranolol transformed the management of infantile hemangiomas by causing involution of vascular tumors that would have otherwise caused severe morbidity to the patient [33]. There are, however, vascular tumors with serious implications for the patient’s health, especially when associated with Kasabach–Merritt phenomenon, and which do not respond adequately to classic treatment with corticosteroids, chemotherapy, or propranolol. Over the last 15 years, a different signaling pathway has become a focus for complex vascular abnormalities. It is well established that the phosphatidylinositol 3-kinase (PI3K)/mammalian target of rapamycin (mTOR) pathway is crucial for tissue development and angiogenesis. Mutations in tumor suppressors upstream of mTOR lead to unregulated mTOR signaling, vascular abnormalities, and illnesses associated with tissue expansion (tuberous sclerosis, lymphangioleiomatosis) [34].

Thrombocytopenia and consumptive coagulopathy are the hallmarks of the KMP, which is triggered by aberrant endothelial cell development in pediatric vascular tumors, such as tufted angioma or kaposiform hemangioendothelioma (KHE). According to earlier studies, the population prevalence of KHE/tufted angioma is 0.91 per 100,000, with up to 60% of neonates developing KMP and 71% progressing to KMP throughout infancy [35]. KMP has a mortality rate of 10% to 40% and is caused by rapid tumor growth and invasion, compression of important structures, or significant bleeding. In children with KHE, independent risk factors for KMP include age of onset ≤1-month, deeper lesions, non-extremity sites, and lesions >60 mm [36]. Vincristine, propranolol, and corticosteroids have been suggested as conservative treatments for KHE since the therapy can be challenging. Nevertheless, some individuals do not respond to these medications, necessitating elaborate strategies that involve radiation, resection, and embolization in addition to medicine [37].

Recent studies have shown that sirolimus, either as monotherapy or in combination with prednisolone, is highly effective in the treatment of kaposiform hemangioendothelioma complicated by KMP, including in neonatal patients [35,38,39]. Nevertheless, there is still little proof that sirolimus treatment for KMP in newborns and babies is effective. Nakammura et al. reported the case of a female neonate having a tumor in the right anterior chest that developed into respiratory distress and congestive heart failure, with KMP, treated with sirolimus alone, 0.06 mg/day. Sirolimus administration resulted in a stable tumor after a year, decreased bleeding, and a reduction in mass without any negative side effects. Thus, lower doses of sirolimus (3–5 ng/mL serum levels) may be less harmful for newborns and may be useful for treating KMP patients [38].

Another case of neonatal KHE (a solid, violaceous swelling that occupies the right cervicothoracic area) with KMP presenting as severe airway obstruction at birth was reported by Shin et al. [40]. Intravenous immunoglobulin (IVIG), corticosteroids, platelet and plasma transfusions, and antithrombin III replacement were the first treatments. Due to gastrointestinal effects, vincristine was rapidly stopped. After starting sirolimus medication on day 14, platelets quickly recovered. Significant tumor regression was noted after three months, and the patient was stable at 16 months.

Sirolimus can even be administered to recently BCG (Bacillus Calmette–Guérin)-vaccinated newborns despite rapamycin immunosuppressive effects if the patient exhibits a KHE with KMP, as Zheng et al. proceeded in a newborn whose initial line of treatment for KMP failed [41]. To avoid infections, prophylactic trimethoprim-sulfamethoxazole was also administered to this neonate.

According to the study of Ji et al., one of the most frequent complications of KHE, whether associated or not with KMP, is chronic lymphedema, which can occur independently of sirolimus treatment. Patients should be cautiously watched for this incapacitating consequence if they have substantial and mixed KHE involving extremities [39].

A series of 4 neonatal cases presented by Cîrstoveanu et al. showed a favorable evolution of all cases treated with an association of sirolimus and propranolol, with only side effect being hypertriglyceridemia [42]. Those four newborns were diagnosed with distinct vascular abnormalities as follows: congenital lymphatic malformation (CLM) on the left hemithorax with the involvement of the left upper limb; complicated congenital hepatic hemangioma in the first 7 weeks of life; KHE of the left lower limb complicated with KMP, and another one most probably with diffuse capillary malformation with overgrowth. Sirolimus was first administered at a dose of 0.45–0.5 mg/m^2^, with dosages modified based on plasmatic levels of 7–12 ng/mL. Depending on tolerability, propranolol dosages were raised from 0.5–1.0 mg/kg/day to 3.0 mg/kg/day. After two months, each patient had a noticeable decrease in the mass’s size, an improvement in the tumor’s appearance, or even calcification in the hepatic vascular tumor.

In Table 1 we presented the main results of some relevant articles or case reports on newborns or fetuses treated with sirolimus, with an accent on dosage and target serum concentration.

## 5. Sirolimus Treatment Side Effects

A thorough risk-benefit analysis and close monitoring are necessary when using off-label medication like sirolimus in children with VAs. Children using sirolimus for VAs should be closely monitored since they may be at increased risk for adverse reactions. The most frequent side effects observed in 18 trials included in the Wiegand et al. systematic review were infections such as cellulitis, neutropenia, and hyperlipidemia [15].

Sirolimus was shown to improve bleeding, oozing, self-assessed efficacy, quality of life, and pain, particularly for combined malformations, in a multicenter, open-label, observational-phase randomized clinical trial involving 59 children aged 6 to 18 years with a slow-flow vascular malformation. 56 patients had 231 adverse events while taking sirolimus (5 significant adverse events, none were life-threatening). Oral ulcers were the most common adverse event, occurring in 29 patients (49.2%) [3]. One case of viral ileitis, one case of septic arthritis of a toe, one case of sepsis caused by *Pseudomonas aeruginosa*, one case of severe constipation, and one case of deep vein thrombosis of the leg affected by Klippel–Trenaunay syndrome occurred six weeks after starting sirolimus treatment. Of the 231 adverse events that occurred during the interventional period, 89 were likely related to sirolimus treatment [3].

Grenier et al. retrospectively evaluated 136 patients who received oral sirolimus treatment at 0.8 mg/m^2^ per dose twice daily for VAs. From them, 75 patients (55%) developed mucocutaneous adverse events: stomatitis, acneiform eruption, worsening skin ulceration, or eczematous eruption. The median (range) period of treatment was 2.5 years, the median dose of sirolimus was 2 mg, and the median time to adverse events was 2 months after starting sirolimus. When adverse events occurred, the median sirolimus trough level in these patients was 6.7 ng/mL (2–11.8 ng/mL) [43]. Most sirolimus side effects were managed with conservative measures or by reducing the medication dosage.

Numerous studies, however, point out that the current standard dose (0.8 mg/m^2^ twice daily) and blood concentration are high for neonates, which can easily result in stomatitis, gastrointestinal reactions, fever, rash, pain, and even potentially fatal *Pneumocystis carinii* pneumonia [44]. The hepatic enzyme CYP3A4, which metabolizes sirolimus, is little-expressed in neonates after birth and gradually increases during the first year of life. As a result, sirolimus’s bioavailability and half-life in infants can vary. That is why, for neonates, Czechowicz et al. recommended a target serum concentration range of 4–10 ng/mL for sirolimus [45]. Harbers et al. demonstrated that sirolimus was still effective in treating KHE and KMP patients with very low serum concentrations (2–6 ng/mL) without causing significant side effects [46]. Combining low-dose sirolimus with prednisolone was a very good option for the neonate with KHE and KMP reported by Cheng et al. [35].

## 6. Discussions

Sirolimus (rapamycin), an mTOR inhibitor, was originally approved by the U.S. Food and Drug Administration for the prevention of kidney transplant rejection; however, due to its molecular target, it has also emerged as an important therapeutic agent for the treatment of complex VAs. Sirolimus has emerged as a safe and effective therapeutic option; however, current dosing recommendations typically apply to patients aged six or seven months and older. There is a clear need for evidence-based dosing guidelines for very young infants, in whom hepatic metabolism of sirolimus is reduced.

In contrast to high-flow vascular malformations, for which sirolimus appears to be less effective as a treatment, slow-flow vascular malformations demonstrated encouraging, but inconsistent, results in prior observational reports, with varying sirolimus dosages and outcomes [8,47].

Observation, physical therapy, sclerotherapy, full or partial resection, and medications like mammalian target of rapamycin (mTOR) inhibitors are all used in the management of VMs and LMs [48,49]. Although LMs typically enlarge slowly, in proportion to a patient’s somatic growth, recurrent infections, trauma, or intracystic hemorrhage may lead to sudden expansion requiring medical or interventional treatment. In addition, antenatally detected LMs of the head and neck may cause acute airway obstruction at birth, necessitating urgent management, and sirolimus has been shown to be effective in such cases [48]. Pharmacologic treatment with various medications is considered for the treatment of VAs, and sirolimus appears to be a promising systemic treatment that has been found to decrease lymphangiogenesis, exhibit antiangiogenic and antiproliferative properties, and may be helpful in the therapy of certain conditions [15].

Following an analysis of all available studies reporting the use of sirolimus in neonates with VAs, we found a lack of consensus regarding indications for treatment initiation, optimal dosing regimens, and duration of therapy. In most studies, neonates were analyzed as part of broader pediatric cohorts that included patients up to 18 years of age, rather than as a distinct population. Moreover, very few investigations specifically focus on neonates treated with sirolimus and evaluate treatment outcomes from a neonatal subgroup perspective. The available evidence largely consists of isolated case reports describing sirolimus use in neonates for various VAs, most commonly lymphatic malformations or KHE; in some instances, sirolimus was administered in combination with propranolol, corticosteroids, or other therapeutic interventions. Nevertheless, across both clinical studies and case series and reports involving fetuses or neonates, sirolimus is generally reported to be effective and tends to be well tolerated, with adverse effects that are minimal and reversible.

However, not all patients demonstrate a therapeutic response. Therefore, identifying predictors of responsiveness to sirolimus and stratifying patients who are most likely to benefit from this therapy are of critical clinical importance. The genetics and expression levels of VEGF-C and VEGFR-3, as well as key components of the mTOR signaling pathway (PI3K/AKT/mTOR), including S6K1, may represent potential biomarkers of treatment efficacy [47,50]. For neonates, lowering the blood concentration range can be appropriate to prevent serious, life-threatening infections. The sirolimus dosage should be adjusted based on the child’s age and weight.

This review of the literature has certain limitations. We observed a lack of consistency and thoroughness in the reporting, and critical information about newborns was frequently absent. Many researchers used qualitative rather than quantitative outcome measures, and the results were presented at various stages in time; adverse reactions to sirolimus treatment, if present, were also lacking. Retrospective studies and case reports are common and restrict how broadly the findings can be applied, raising the possibility of reporting bias.

Future directions: Major risks—such as obstructive respiratory failure or KMP- as well as disfiguring scars following surgery or sclerotherapy for VAs—can be minimized through accurate diagnosis and prompt initiation of appropriate therapy. There is an urgent need for future research examining the efficacy and safety of sirolimus treatment in fetuses and neonates. Research into genes and mechanisms involved in vascular angiogenesis and lymphangiogenesis must continue, and clinical study designs have to specifically address the physiological characteristics of the neonatal population. In addition, the structure of multidisciplinary teams providing care for VAs, as well as protocols used to monitor the efficacy and safety of sirolimus therapy, should be regularly updated with greater emphasis on the unique needs of neonates.

Clinical recommendations:

✓ Fetuses and neonates with life-threatening VAs, such as LMs causing upper airway obstruction, bleeding MVs, or KHE associated with KMP, who cannot benefit from traditional treatments such as sclerotherapy, laser therapy, or surgery, sirolimus alone or in combination with prednisolone should be considered as a treatment option.
✓ During sirolimus therapy in neonates, regular monitoring of liver function and hematologic parameters is essential, with dose adjustments made to achieve a target serum concentration of 2–10 ng/mL to minimize adverse effects and optimize therapeutic efficacy.
✓ Most sirolimus side effects can be managed with conservative measures or by reducing the medication dosage.
✓ Given that current research primarily focuses on short-term efficacy and safety, clinicians should remain vigilant regarding the long-term effects and potential risks of sirolimus treatment initiated during the neonatal period and ensure timely, structured follow-up.

## 7. Conclusions

For fetuses and neonates with large, complex VAs, sirolimus alone or in combination with other therapeutic options appears to be a successful treatment without serious adverse events. During sirolimus therapy in neonates, regular monitoring of liver function and hematologic parameters is essential, with dose adjustments performed as needed to minimize adverse effects and optimize therapeutic efficacy. A multidisciplinary team should decide whether to treat a newborn with sirolimus, and parents should be well informed about any possible side effects. Well-designed randomized controlled or observational studies should be conducted to assess sirolimus’ efficacy and safety in neonates.

## Figures and Tables

**Table 1:** Sirolimus treatment for complex fetal and neonatal vascular anomalies—evidence from the literature.

Vascular Anomaly	Sirolimus Dose and Target Concentration	Effectiveness	Adverse Effects	Study
Cervicofacial Cystic Lymphatic Malformation in a neonate	Oral sirolimus, 0.1 mg/kg/day, twice daily, for the first month. Then, 0.02–0.15 mg/kg/day. In combination with 3 additional percutaneous sclerotherapies. Target concentration: 12–20 ng/mL (tested twice a week) for the first month. Then, 5.7–19 ng per mL	From day 0 to 14 months of life, good evolution was observed from the first month, with “the persistence of only one microcyst” at 7 months	At 7 months, mild dyslipidemia with a moderate increase in cholesterol and triglyceride levels (spontaneously normalized at 9 months)	Case report—Honnorat et al., 2020 [19]
Cervicofacial Cystic Lymphatic Malformation in 7 neonates	Oral sirolimus, 0.8 mg/m^2^ per dose, twice daily. 2 patients—additional subtotal surgical resection and one tracheostomy. Target concentration: 4–12 ng/mL	Median follow-up 32 months; median treatment 12 months. 1 patient—complete resolution of the malformation, 1—complete resolution of symptoms, and 5—partial resolution of the malformation with significant improvement in respiratory conditions.	Toxicity was not observed	Retrospective study—Triana et al., 2019 [21]
Cervicofacial Cystic Lymphatic Malformation in a 30-week fetus	Maternal: oral sirolimus 15 mg load, then 5 mg once daily. Target concentration: Maternal 5–15 ng/mL	Rapid reduction in fetal cervicofacial mass size	No adverse effects to mother or fetus/neonate	Case report—Livingston et al., 2021 [23]
Cervicofacial Cystic Lymphatic Malformation in 105 children (newborn—17 years)	Oral sirolimus, 0.8 mg/m^2^ per dose, twice daily. Target concentration: 10–15 ng/mL and 5–15 ng/mL	>91% of patients responded to sirolimus treatment, which lasts from 6 months to 4 years	Hyperlipidemia, neutropenia, cellulitis and infections	Systematic review—Wiegand et al., 2022 [15]
Extensive lymphatic–venous malformation of the cervical and thoracic regions in a neonate	Started oral sirolimus, 0.8 mg/m^2^ per dose, twice daily, then reduced dose to 0.4 mg/m^2^ daily. Later underwent limited resection, cyst aspiration and VAC therapy. Target concentration: 4–13 ng/mL	After several weeks, the mass appeared subjectively smaller in size	No side effects	Case report—Avila et al., 2025 [27]
Blue rubber bleb naevus syndrome in a 38-day-old preterm baby	Started oral sirolimus, average dose of 0.95 mg/m^2^/day (0.1 mg/kg/day), then adjusted dose to 0.81 ± 0.13 mg/m^2^/day (0.03 ± 0.01 mg/kg/day) Target concentration: 10–15 ng/mL (evaluated every 1–3 months)	Hemoglobin levels recovered to normal, and the lesion shrank (marked regression)	No adverse drug reactions during the 28-month treatment course	Case report—Yang et al., 2021 [28]
Blue rubber bleb nevus syndrome with Kasabach–Merritt phenomenon in a neonate	Oral sirolimus, 0.8 mg/m^2^/day for 6 months and oral prednisone tablets at 2 mg/kg/day, then 1 mg/kg/day (for 2 months). Target concentration: 5–15 ng/mL	The patient did not receive a blood transfusion again, and there were no signs of KMP recurrence. MRI showed that the lesions were significantly smaller than before.	No significant adverse drug reactions were observed.	Case report—Pi et al., 2023 [29]
Congenital capillary–lymphatic–venous malformations in 2 fetuses	From the 32nd and 33rd weeks of pregnancy until birth, the mothers took oral sirolimus (for 11 and 31 days, respectively) at doses ranging from 2 to 6 mg/day Target concentration trough whole-blood level of 7–12 ng/mL (mother) and umbilical cord arterial blood levels of 3.8–6.4 ng/mL	Both fetuses showed good therapeutic results from prenatal sirolimus: one had less intralesional hemorrhage, while the other had a marked reduction in CLVM size. Both infants received postnatal sirolimus for 9 months and 20 months.	No negative effects on the mothers or the children	Case report—Klosowska et al., 2025 [30]
Klippel–Trenaunay syndrome associated with chylothorax in a neonate	Oral sirolimus, 0.8 mg/m^2^ twice daily, for 7 months Target concentration: 10–15 ng/mL	At the age of seven months, chylothorax had not returned	Elevated gamma-glutamyl transferase was the only side effect	Case report—Kuo et al., 2022 [32]
Kaposiform hemangioendothelioma with KMP in a neonate	Oral sirolimus, 0.06 mg/day Target concentration: 3–5 ng/mL	Stable tumor after a year, decreased bleeding, and a reduction in mass (right anterior chest)	No side effects	Case report—Nakamura et al., 2024 [38]
Lymphatic malformation of hemithorax, Congenital hepatic hemangioma, KHE complicated with KMP, Diffuse capillary malformation with overgrowth in 4 neonates	Oral sirolimus, 0.45–0.5 mg/m^2^ In combination with propranolol 0.5–1.0 mg/kg/day to 3.0 mg/kg/day Target concentration: 7–12 ng/mL	After two months, each patient had a noticeable decrease in the mass’s size, an improvement in the tumor’s appearance, or even calcification in the hepatic vascular tumor	Hypertriglyceridemia	Series of cases -Cîrstoveanu et al., 2021 [42]

KMP—Kasabach–Merritt phenomenon; KHE—Kaposiform hemangioendothelioma.

## Data Availability

No new data were created or analyzed in this study.
